# *In vivo* loss of tumorigenicity in a patient-derived orthotopic xenograft mouse model of ependymoma

**DOI:** 10.3389/fonc.2023.1123492

**Published:** 2023-03-03

**Authors:** Jacqueline P. Whitehouse, Hilary Hii, Chelsea Mayoh, Marie Wong, Pamela Ajuyah, Paulette Barahona, Louise Cui, Hetal Dholaria, Christine L. White, Molly K. Buntine, Jacob Byrne, Keteryne Rodrigues da Silva, Meegan Howlett, Emily J. Girard, Maria Tsoli, David S. Ziegler, Jason M. Dyke, Sharon Lee, Paul G. Ekert, Mark J. Cowley, Nicholas G. Gottardo, Raelene Endersby

**Affiliations:** ^1^ Brain Tumour Research Program, Telethon Kids Institute, Nedlands, WA, Australia; ^2^ Centre for Child Health Research, University of Western Australia, Nedlands, WA, Australia; ^3^ Children’s Cancer Institute, Lowy Cancer Research Centre, UNSW Sydney, Kensington, NSW, Australia; ^4^ School of Clinical Medicine, University of New South Wales (UNSW) Sydney, Kensington, NSW, Australia; ^5^ Department of Paediatric and Adolescent Oncology/Haematology, Perth Children’s Hospital, Nedlands, WA, Australia; ^6^ Division of Paediatrics, University of Western Australia Medical School, Nedlands, WA, Australia; ^7^ Genetics and Molecular Pathology Laboratory, Hudson Institute of Medical Research, Clayton, VIC, Australia; ^8^ Department of Molecular and Translational Science, Monash University, Clayton, VIC, Australia; ^9^ Division of Genetics and Genomics, Victorian Clinical Genetics Services, Parkville, VIC, Australia; ^10^ Medical School of Rbeirão Preto (FMRP-USP), University of São Paulo, São Paulo, Brazil; ^11^ Clinical Research Division, Fred Hutchinson Cancer Research Center, Seattle, WA, United States; ^12^ Ben Towne Center for Childhood Cancer Research, Seattle Children’s Research Institute, Seattle, WA, United States; ^13^ Kids Cancer Centre, Sydney Children’s Hospital, Randwick, NSW, Australia; ^14^ Department of Neuropathology, PathWest Laboratory Medicine, Royal Perth Hospital, Perth, WA, Australia; ^15^ Pathology and Laboratory Medicine, University of Western Australia, Nedlands, WA, Australia; ^16^ Department of Neurosurgery, Perth Children’s Hospital, Nedlands, WA, Australia; ^17^ Murdoch Children’s Research Institute, Royal Children’s Hospital, Parkville, VIC, Australia; ^18^ Cancer Immunology Program, Peter MacCallum Cancer Centre, Melbourne, VIC, Australia; ^19^ The Sir Peter MacCallum Department of Oncology, University of Melbourne, Parkville, VIC, Australia

**Keywords:** ependymoma, posterior fossa, patient-derived, xenograft, molecular, pediatric cancer, brain cancer, mouse model

## Abstract

**Introduction:**

Ependymomas (EPN) are the third most common malignant brain cancer in children. Treatment strategies for pediatric EPN have remained unchanged over recent decades, with 10-year survival rates stagnating at just 67% for children aged 0-14 years. Moreover, a proportion of patients who survive treatment often suffer long-term neurological side effects as a result of therapy. It is evident that there is a need for safer, more effective treatments for pediatric EPN patients. There are ten distinct subgroups of EPN, each with their own molecular and prognostic features. To identify and facilitate the testing of new treatments for EPN, *in vivo* laboratory models representative of the diverse molecular subtypes are required. Here, we describe the establishment of a patient-derived orthotopic xenograft (PDOX) model of posterior fossa A (PFA) EPN, derived from a metastatic cranial lesion.

**Methods:**

Patient and PDOX tumors were analyzed using immunohistochemistry, DNA methylation profiling, whole genome sequencing (WGS) and RNA sequencing.

**Results:**

Both patient and PDOX tumors classified as PFA EPN by methylation profiling, and shared similar histological features consistent with this molecular subgroup. RNA sequencing revealed that gene expression patterns were maintained across the primary and metastatic tumors, as well as the PDOX. Copy number profiling revealed gains of chromosomes 7, 8 and 19, and loss of chromosomes 2q and 6q in the PDOX and matched patient tumor. No clinically significant single nucleotide variants were identified, consistent with the low mutation rates observed in PFA EPN. Overexpression of *EZHIP* RNA and protein, a common feature of PFA EPN, was also observed. Despite the aggressive nature of the tumor in the patient, this PDOX was unable to be maintained past two passages *in vivo*.

**Discussion:**

Others who have successfully developed PDOX models report some of the lowest success rates for EPN compared to other pediatric brain cancer types attempted, with loss of tumorigenicity not uncommon, highlighting the challenges of propagating these tumors in the laboratory. Here, we discuss our collective experiences with PFA EPN PDOX model generation and propose potential approaches to improve future success in establishing preclinical EPN models.

## 1 Introduction

Ependymomas (EPNs) are malignant central nervous system (CNS) tumors that can arise in the supratentorial brain, the posterior fossa, or the spinal cord. EPN occurs in both adults and children, but is more frequent in children, accounting for approximately 5% of CNS tumors in children aged 0-14 years (1). There has been little change in the treatment of EPN over recent decades, and current standard of care remains surgical resection of the tumor followed by radiotherapy where appropriate, depending on the patient’s age (2). Survival rates for children with EPN remain inadequate, with a 10-year survival rate of just 67% for those aged 0-14 years (1). Moreover, long-term survivors of the most common pediatric EPN (posterior fossa A (PFA)) experience neuro-cognitive sequelae, as well as other significant late effects as a result of their treatment (3), highlighting the need for more effective and less damaging treatment options for these patients.

In addition to PFA EPN, a landmark study incorporated genetic and epigenetic analyses to identify a further eight molecular subgroups of EPN (4), with the recent description of a tenth subgroup (5, 6), which have been incorporated into the most recent edition of the World Health Organization classification of CNS tumors (7). Of these subgroups, PFA EPN is the most common subgroup affecting infants and young children and carries a dismal prognosis, with 10-year overall survival rates of approximately 56% (4). When patients are stratified by extent of resection, 10-year overall survival rates plummet further to just 32.7-45.1% for patients with subtotal resection (8). PFA EPN are considered epigenetically-altered tumors and are frequently characterized by loss of histone H3 lysine 27 tri-methylation and overexpression of *EZHIP* (also known as *CXorf67*) (9, 10). No recurrent genetic drivers have been identified for PFA EPN (11), however gain of chromosome 1q and loss of chromosome 6q have been identified as poor prognostic indicators (4, 10, 12).

Molecular classification in other brain tumor types, such as medulloblastoma, has demonstrated the value and importance of clinically stratifying and treating CNS tumors based on molecular features (13). In order to best use this information in the preclinical translational space for PFA EPN, we need to develop representative laboratory models to facilitate the identification and testing of new treatments for this disease (14, 15). The lack of clear genetic drivers for PFA EPN precludes the ability to generate genetically engineered mouse models, and thus we currently rely heavily on the establishment of patient-derived orthotopic xenograft (PDOX) models to represent this cancer in the laboratory. However, the development of these models is a challenging task, requiring specialized skills and significant time and resource input for a relatively low chance of engraftment success (16–19). Here, we describe the establishment of a PDOX model of PFA EPN that persisted for two passages *in vivo* before losing tumorigenicity. The challenges of PFA EPN PDOX model generation are discussed, as well as potential approaches that may help drive success in the establishment and propagation of these models in the future.

## 2 Materials and methods

### 2.1 Human samples

The parents/guardians of the patient gave their informed consent before the donation of the tumor tissue for research purposes and for retrospective research access to relevant medical records and previously obtained pathology samples. Written informed consent was obtained from the minor’s legal guardian for the publication of any potentially identifiable images or data included in this article. The study was conducted in accordance with the Declaration of Helsinki, and the protocol was approved by the Ethics Committee of the Child and Adolescent Health Service, Western Australia (HREC: 1769/EP (PRN 0000002372) A Perth Children’s Hospital Oncology Protocol for Collecting and Banking Paediatric Research Specimens; approved 21/08/2003).

### 2.2 Implantation of patient tumor cells and *In vivo* serial transplantation

Animal experiments were approved by the Animal Ethics Committee of the Telethon Kids Institute and performed in accordance with Australia’s Code for the Care and Use of Animals for Scientific Purposes (AEC#263 approved 1/9/2013, AEC#300 approved 18/4/2016, AEC#362 approved 24/4/2020). Immunodeficient BALB/c nude mice were purchased from the Animal Resources Centre (Murdoch, Western Australia, Australia) and J:NU mice were obtained from The Jackson Laboratory (Bar Harbor, Maine, USA). Implantation of tumor cells was performed as previously described (20). Specifically, approximately 1 hour following the patient’s final surgical procedure, tumor tissue (fourth surgical sample) from the patient (ID 801806) was mechanically dissociated, filtered through a 100 μm cell strainer, and suspended in Matrigel (BD Biosciences, San Jose, California, USA). Animals received general anesthesia and pre-operative analgesia (ketamine 100mg/kg intraperitoneally, medetomidine 1mg/kg intraperitoneally) and post-operative analgesia (0.4mg/ml ibuprofen in drinking water for five days). Cells (approximately 10^6^ per mouse in 2μl) were implanted into the cortex (approximately -0.45mm from bregma at a depth of 3mm; n=3) or cerebellum (approximately -6.3mm from bregma at a depth of 2mm; n=2), of five 8-week-old mice using a Hamilton syringe. The implantation process took approximately 5 minutes per mouse. Upon tumor-related morbidity, the brain was bisected at the implantation site and one half of the brain containing the tumor was kept for histology. The remaining tumor was removed, dissociated and reimplanted into the cortex of successive recipients as described above (referred to as PDOX TK-EPN862). At autopsy, no evidence of leptomeningeal spillage of tumor cells from the implantation procedure was observed, nor was there evidence of leptomeningeal metastasis of the tumor.

### 2.3 Histochemical staining

Tissue samples were fixed in 4% paraformaldehyde in phosphate buffered saline or neutral buffered formalin for 24 hours and embedded in paraffin. Patient and mouse PDOX tissue sections (5 µm) underwent microwave antigen retrieval in a citrate buffer before immunohistochemistry (IHC) using the following antibodies and dilutions: Olig2 (Millipore, Burlington, MA, USA, MABN50; 1:200), Tri-methyl-histone H3 (K27) (Cell Signaling, Beverly, MA, USA, 9733; 1:200), GFAP (Sigma Aldrich, St Louis, MO, USA, G3893-2ML; 1:200), Ki67 (Cell Signaling, 9027; 1:400), p53 (Cell Signaling, 2527; 1:160), synaptophysin (Cell Signaling, 36406; 1:200), EMA (Dako, Santa Clara, CA, USA, M0613; 1:100) and EZHIP (Sigma Aldrich, HPA004003-25UL; 1:200). Sections were incubated with species-specific biotinylated goat anti-IgG secondary antibodies, followed by detection with an Elite ABC kit and NovaRED peroxidase substrate, then counterstained with Gill’s hematoxylin according to manufacturer’s instructions (Vector Laboratories, Burlingame, California, USA). Hematoxylin and eosin (H&E) staining was performed as per standard protocols using a Leica Autostainer XL.

### 2.4 DNA and RNA extraction

Genomic germline DNA was prepared from peripheral blood mononuclear cells using a QIAamp DNA Mini Kit (Qiagen, Hilden, North Rhine-Westphalia, Germany, 51304) as per the manufacturer’s instructions for DNA extraction from lymphocytes. Genomic tumor DNA and RNA were prepared from fresh frozen patient and PDOX tumor samples using an AllPrep DNA/RNA Mini Kit (Qiagen, 80204) according to the manufacturer’s instructions. DNA quality was determined by gel electrophoresis and spectrophotometry (Nanodrop, Thermo Fisher Scientific, Waltham, MA, USA), and quantified using fluorometry (Qubit, Life Technologies, Waltham, MA, USA, Q32851). RNA quality and quantity were determined using the LabChip GX nucleic acid analyzer (Perkin Elmer, Waltham, MA, USA) (performed by the Australian Genome Research Facility, Perth, Western Australia, Australia).

### 2.5 Methylation array

Genomic DNA (500–1000 ng) was treated with sodium bisulphite using the EZ DNA methylation kit (Zymo Research, Irvine, CA, USA) and bisulphite conversion was confirmed by methylation specific PCR as described previously (21, 22). Quantification of DNA methylation was performed at the Australian Genome Research Facility (Melbourne, Victoria, Australia) using the Human Methylation EPIC BeadChip (Illumina, San Diego, CA, USA) run on an Illumina iScan System (Illumina) using the manufacturer’s standard protocol. Raw idat files were uploaded to an online DNA methylation-based classification of CNS tumors platform (www.molecularneuropathology.org, version 11b4 and version 12.5) (23) and basic copy number variant profiles from methylation array data analyzed using the output generated from this classifier (24). Fisher’s exact test was performed using GraphPad Prism software (version 9.4.0) to determine statistical significance between 1q gain and PDOX generation success.

### 2.6 Whole genome sequencing

Whole genome sequencing (WGS) data obtained from the patient germline and tumor DNA samples were analyzed as reported in (25). For the PDOX, an additional step to remove mouse reads using BBSplit version June 11 2018 (26) was done prior to the previously described method. Default parameters were used except for ambiguous2 that was set to `toss` in order to conservatively exclude ambiguously mapped reads to either the mouse or human reference genomes. WGS analysis included the identification of somatic single nucleotide variants, short indels, cytogenetic-scale and gene-level copy number and structural variants, as reported in (25). WGS dataset generated by this study are available from the European Genome-Phenome Archive under accession number EGAS00001006843.

### 2.7 RNA sequencing, clustering analysis and expression profiling

RNA sequencing (RNAseq) analysis and expression profiling was performed as reported in (25). Differential expression analysis was conducted using the R package edgeR. Genes were removed if the counts per million was less than 1 in 2 or more samples. Genes were considered significantly differentially expressed with an absolute fold change (|FC|) ≥ 2 and a false discovery rate (FDR) < 0.05. For the differential analysis between cranial metastasis and the PDOX model a further filtration was performed that removed all genes with a counts per million of 0 due to mouse infiltrating reads. Correlation analysis was performed between the different tumors and PDOX using the R package corrplot on the filtered gene set. KEGG pathway enrichment analysis was performed using DAVID with the significant differentially expressed genes from the PDOX comparing the cranial metastasis as input. Transcripts per million (TPM) expression values were used for plotting and for comparing the patient tumor and PDOX model samples against the ZERO cohort, a reference dataset containing high-risk pediatric brain tumors (25). RNAseq dataset generated by this study are available from the European Genome-Phenome Archive under accession number EGAS00001006844.

## 3 Results

### 3.1 Case report

**Figure 1:**
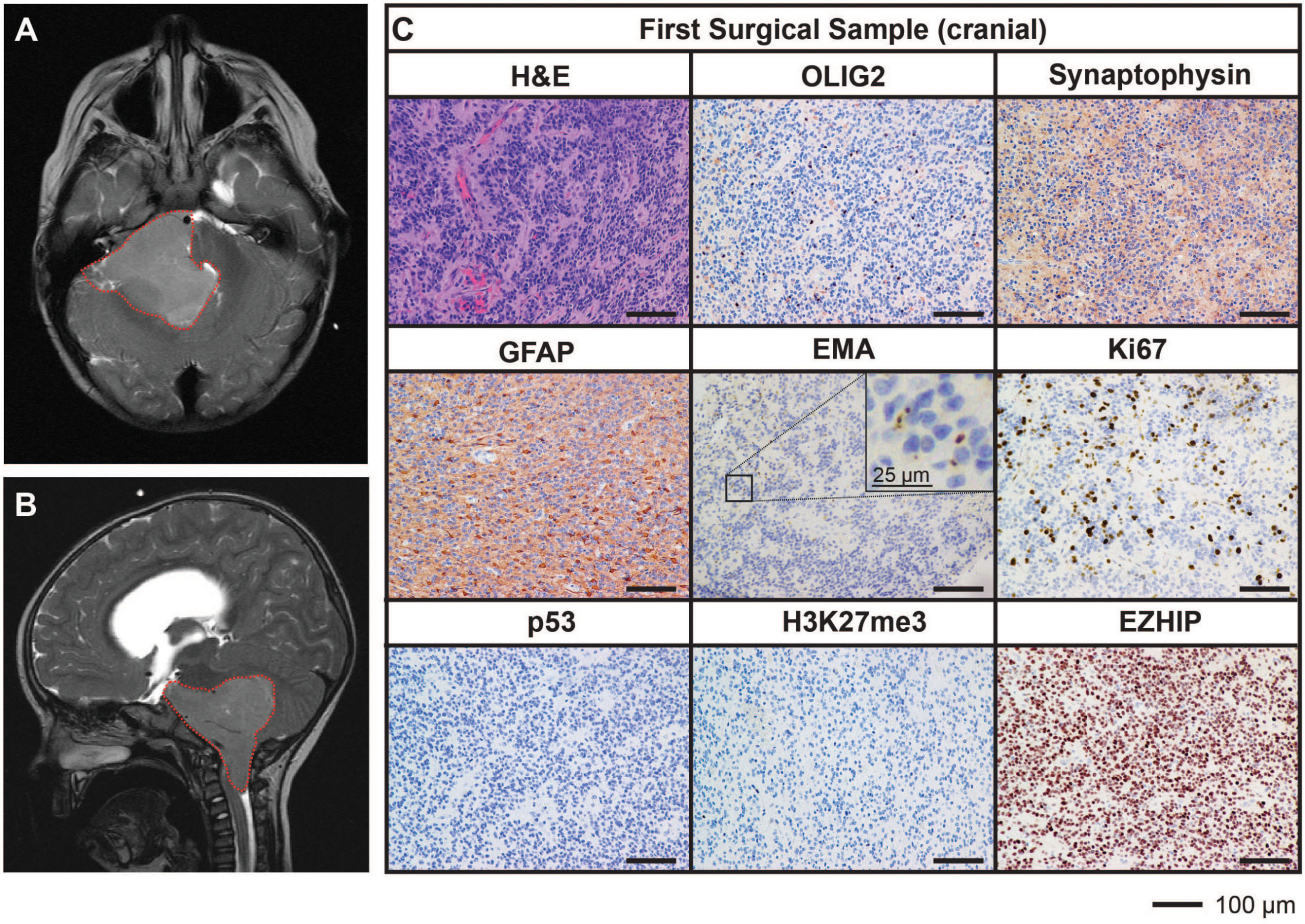
MRI and histological assessments of the first surgical sample (cranial) were consistent with the diagnosis of PFA EPN. **(A, B)** Diagnostic MR images depicting a large posterior fossa mass (*red dotted line*). **(C)** Tumor sections obtained from the first surgery were stained with hematoxylin and eosin **(*H&E*)** or using IHC with the antibodies indicated (*brown*) followed by hematoxylin counterstain (*blue*). Scale bars are as indicated.

A previously well, three-year-old male presented with a three-week history of headache, early morning vomiting, seizures and lethargy. Magnetic resonance imaging (MRI) of the brain revealed the presence of a large posterior fossa mass (71mm by 50mm) within the fourth ventricle and extending into the foramen of Luschka (**Figures 1A, B**). An extraventricular drain was placed to release the pressure followed by subtotal excision of the mass (first surgical sample). Histopathological assessment of the resected mass showed a highly cellular tumor with evidence of widespread perivascular pseudorosettes, moderate nuclear pleomorphism, and multifocal necrosis. Of note, no true ependymal rosettes were identified. The proliferative index, as assessed by Ki67-positivity, was approximately 20% with 9-12 mitotic figures per 10 high power fields of view identified. Tumor cells were predominantly negative for OLIG2 and synaptophysin, positive for GFAP and demonstrated intracytoplasmic dot-like EMA staining. These characteristics are consistent with the diagnosis of WHO grade III EPN with anaplastic features. Immunostaining for p53 was negative. Tumor cells were also negative for histone H3 lysine 27 tri-methylation (H3K27me3) and expressed high levels of EZHIP, consistent with the features of PFA EPN (**Figure 1C**).

Following surgery, the patient suffered from severe posterior fossa syndrome and required intense rehabilitation before being clinically fit to receive radiotherapy. Postoperative imaging confirmed a residual mass (12mm by 8mm) at the right lateral lower pons with extension over the petrous ridge into the middle cranial fossa. The patient was treated as per the ACNS0831 Children’s Oncology Group study protocol (27). He received two cycles of induction chemotherapy (vincristine, carboplatin, cyclophosphamide and etoposide). Imaging assessment post-induction cycles indicated further progression of the residual tumor (increase to 27mm by 10mm). A further surgical attempt achieved a partial resection. Histopathological assessment revealed the residual mass retained the features of the original tumor, however no tissue sample was available from this resection for research. The patient received 59.4 Gy of focal radiotherapy followed by four cycles of maintenance chemotherapy (vincristine, cisplatin, cyclophosphamide and etoposide).

**Figure 2:**
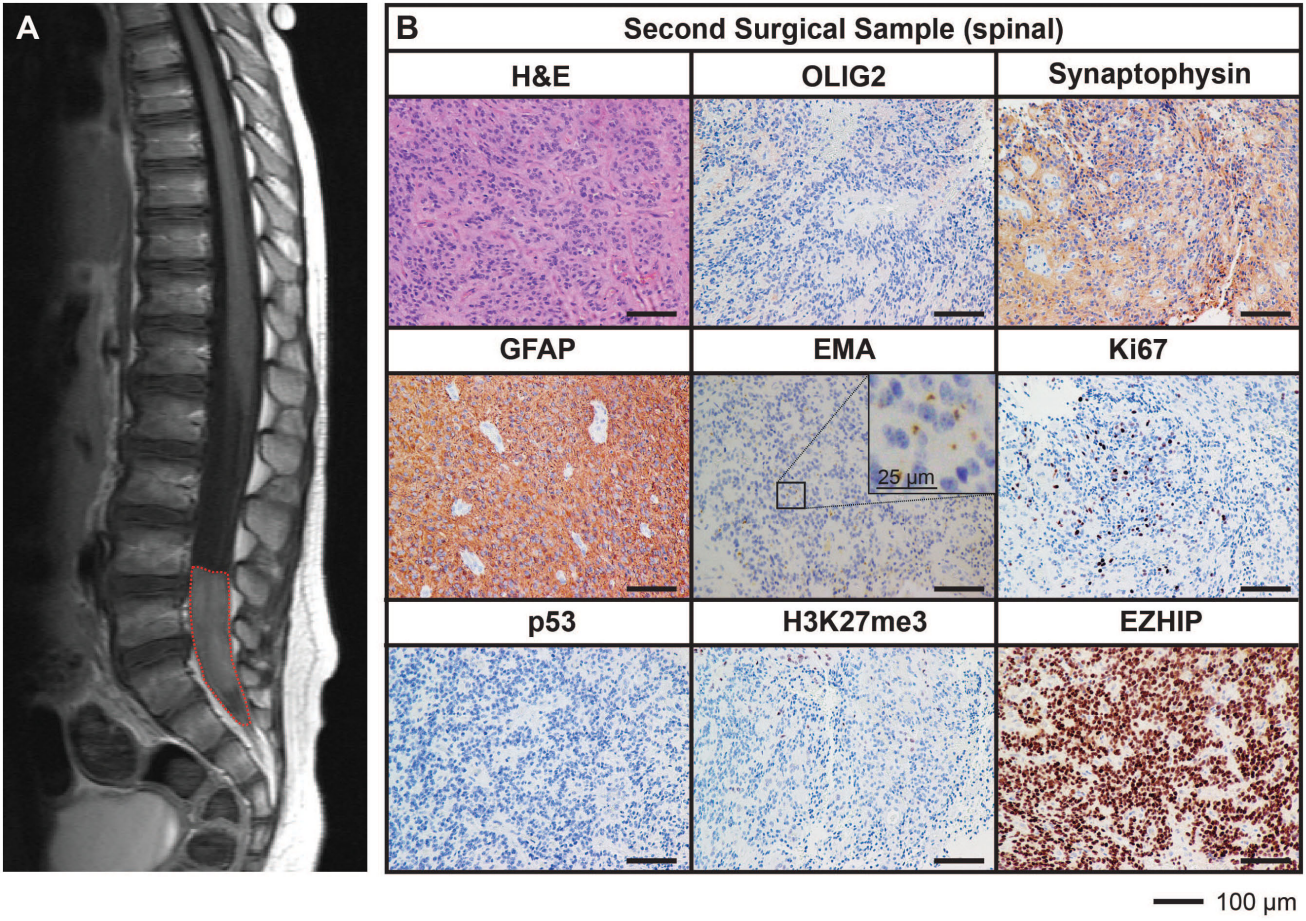
MRI and histological assessments of the second surgical sample (spinal) were consistent with the lesion being a metastasis of the primary PFA EPN. **(A)** MRI depicting a spinal metastasis in the terminal thecal sac (*red dotted line*). **(B)** The tumor tissue was stained with H&E or using IHC with the antibodies indicated (*brown*) followed by hematoxylin counterstain (*blue*). Histological findings were consistent with the features of the primary tumor. Ki67 proliferative index was estimated to be 25%. Scale bars are as indicated.

After being in remission for 12 months following the completion of treatment, surveillance imaging revealed the presence of a solitary large drop metastasis in the terminal thecal sac between L4-S2 (**Figure 2A**) with stable residual intracranial disease. Complete resection of the spinal lesion was performed (second surgical sample), followed by 36 Gy craniospinal irradiation with 14.4 Gy focal boost. The histological features of the metastasis were consistent with the primary lesion (**Figure 2B**).

**Figure 3:**
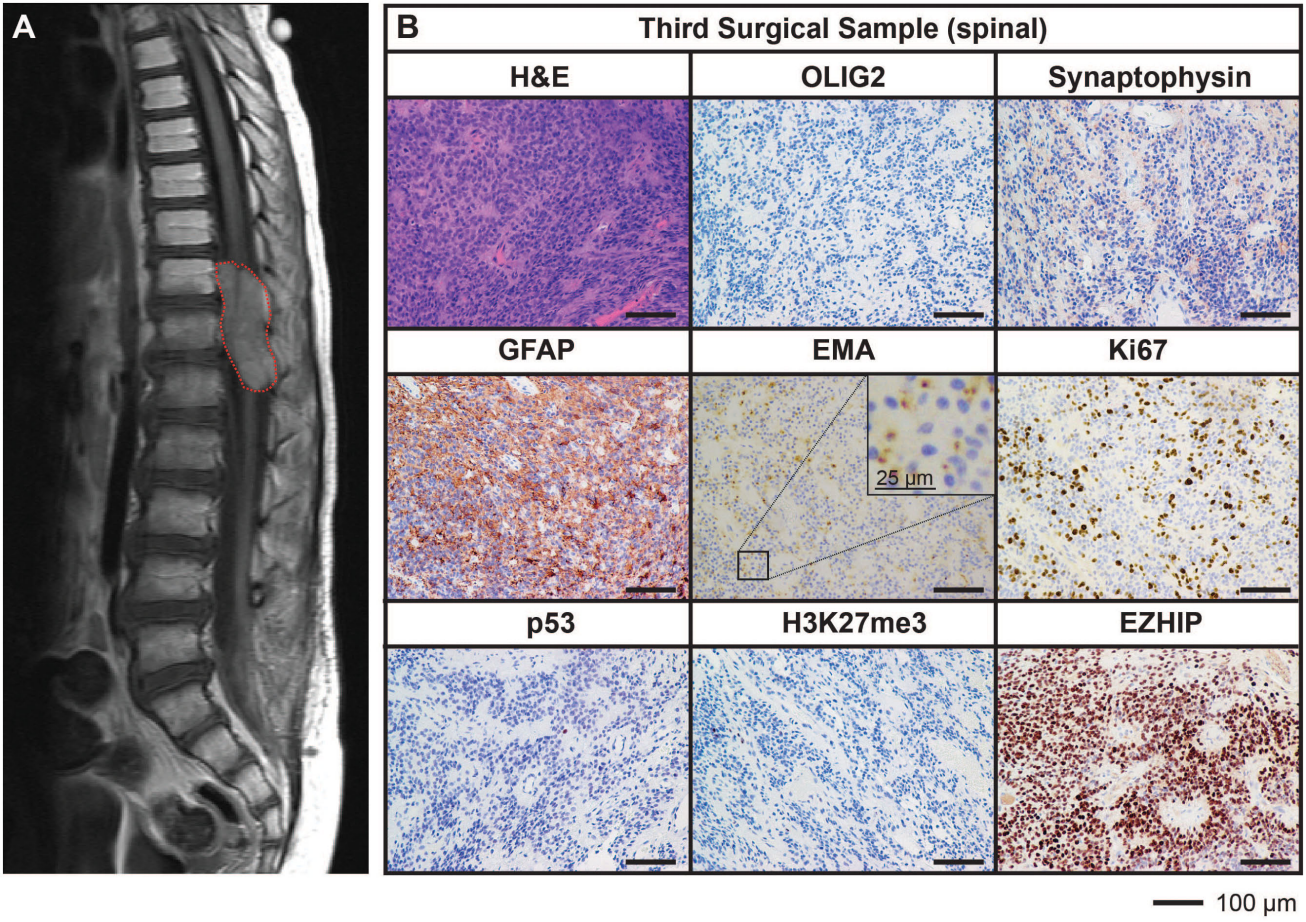
MRI and histological assessments of the third surgical sample (spinal) were consistent with the lesion being an additional metastatic tumor arising in the spine from the initial PFA EPN. **(A)** MRI depicting a large spinal metastasis (*red dotted line*). **(B)** Tumor tissue was stained with H&E or using IHC with the antibodies indicated (*brown*) followed by hematoxylin counterstain (*blue*). Histological findings were consistent with the features of the primary tumor. Ki67 proliferative index was estimated to be 25%. Scale bars are as indicated.

**Figure 4:**
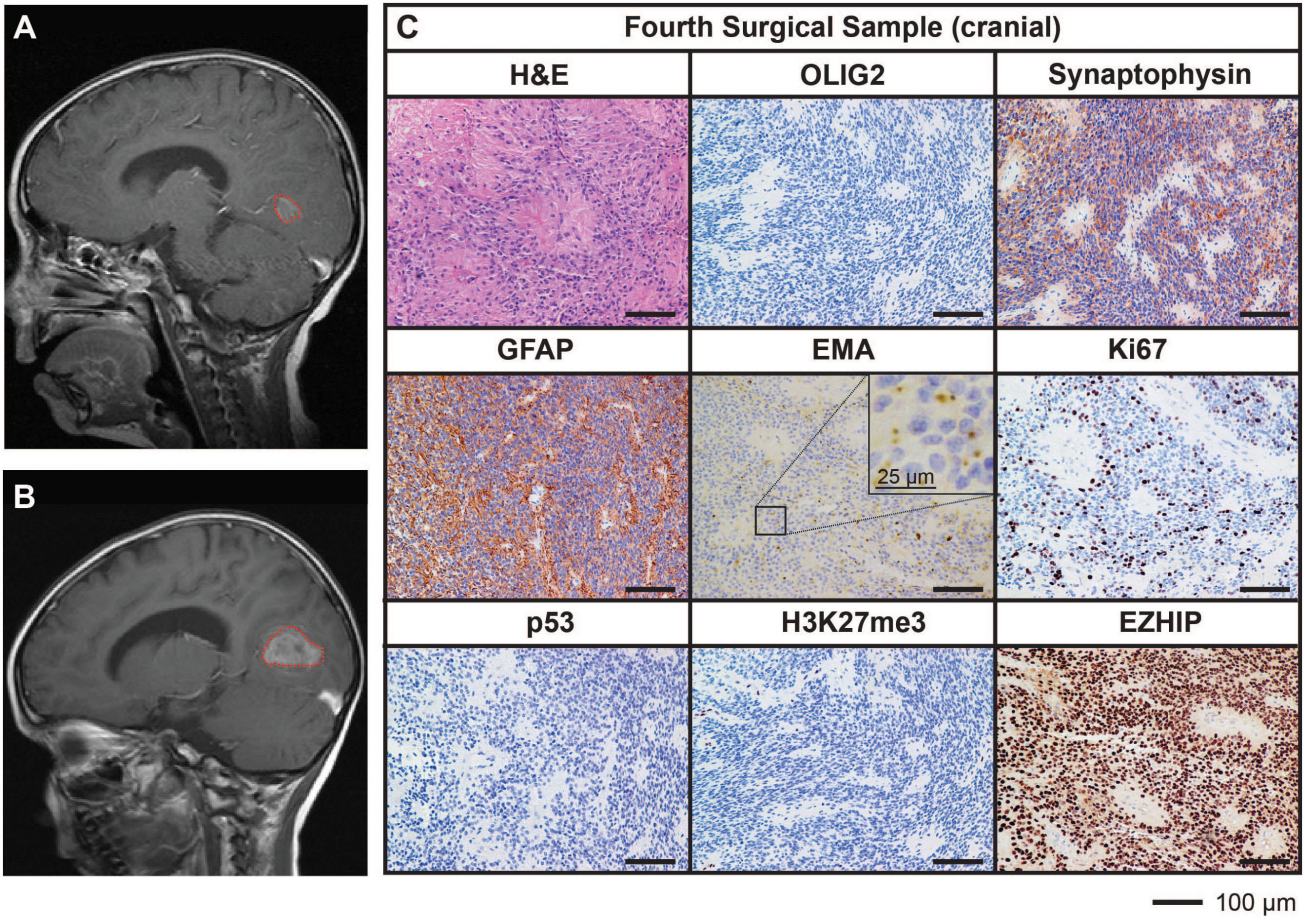
MRI and histological assessments of a cranial metastatic lesion distant from the primary PFA EPN (fourth surgical sample) has features concordant with the initial disease. **(A)** MRI depicting a metastatic tumor in the mesial occipital region (*red dotted line*). **(B)** Progression of the mesial occipital lesion (*red dotted line*) at three months following the scan shown in **(A)**. This tumor required surgical excision, from which a fourth surgical sample was obtained. **(C)** Tumor tissue was stained with H&E or using IHC with the antibodies indicated (*brown*) followed by hematoxylin counterstain (*blue*). Histological findings were consistent with the features of the primary tumor. Ki67 proliferative index was similar to previous samples (25%). Scale bars are as indicated.

Ongoing surveillance scans 10 months after the completion of craniospinal irradiation detected another metastatic spinal lesion at T12 (**Figure 3A**). The patient then commenced an early phase trial protocol for recurrent malignancies [ACCT007: Rap-CV (28)] involving treatment with rapamycin, cyclophosphamide, and vinorelbine. No response was observed following two cycles of treatment, and the spinal lesion progressed to 50 mm in size. A further metastatic lesion (12 mm) in the mesial occipital region was also discovered at this time (**Figure 4A**). To prevent spinal cord compression, complete excision of the spinal metastasis was performed (third surgical sample), followed by 15 Gy focal radiation. The patient was further treated with fluorouracil according to another early phase clinical trial. The mesial occipital lesion continued to progress (**Figure 4B**) requiring complete resection (fourth surgical sample) followed by a 20 Gy focal boost to the tumor bed. The spinal and cranial lesions were both histologically consistent with previous tumor samples (**Figures 3B**, **4C**). The patient had stable disease for four months, following which leptomeningeal metastases were detected throughout the brain and spinal cord. He was treated with one dose of gemcitabine according to an early phase trial treatment without success. The patient was provided with end-of-life care and died a short time later, four years following the primary diagnosis. A summary of the treatment procedures performed and surgical samples collected is illustrated in **Supplementary Figure 1**.

### 3.2 PFA EPN tumor cells successfully engrafted in mice but serial propagation was unsuccessful

**Figure 5:**
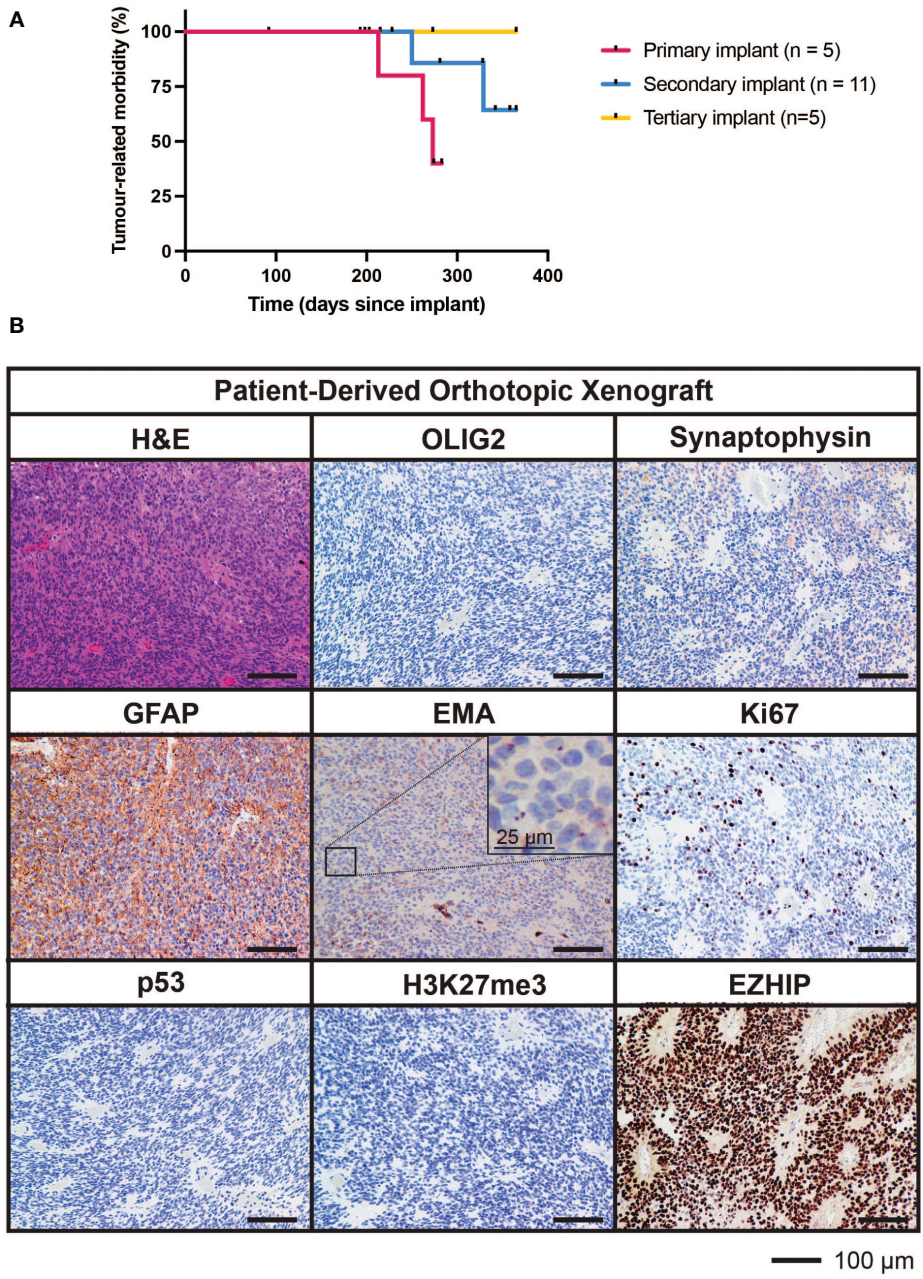
Survival characteristics and histological features of TK-EPN862. **(A)** Time to morbidity in the TK-EPN862 PDOX model. Mice were implanted with tumor cells from the fourth surgical sample (primary implant, *pink*), or serially transplanted with TK-EPN862 cells (secondary implant, *blue*; tertiary implant, *yellow*). Mice euthanized for non-tumor-related reasons were censored (black vertical dash). **(B)** H&E staining (top left) or IHC for the indicated antibodies (*brown*) demonstrate that TK-EPN862 xenografts recapitulated the histological features of the matched surgical sample. Sections are counterstained with hematoxylin (*blue*) and the sizes of the scale bars are indicated.

Tumor cells from the fourth surgical sample were implanted into the brains of five immunodeficient mice. Three of these five mice developed brain tumors (two from cortical implants and one from cerebellar implants) generating a PDOX model termed TK-EPN862. Upon the development of tumor-related morbidity in these animals, tumor tissues were harvested and serially transplanted into the cortex of a further 11 immunodeficient mice. Of these secondary implant recipients, two mice developed a brain tumor. Upon serial implantation of these tumor cells into the cortex of five further mice (tertiary implant recipients), no further tumors grew, resulting in the loss of the PDOX model (**Figure 5A**). Attempts to resurrect the TK-EPN862 model by orthotopically implanting cryopreserved cells were unsuccessful.

The median time to morbidity across all tumor-related deaths was 262 days. Asymptomatic mice were either euthanized for reasons unrelated to tumor growth (such as rectal prolapse) or at the predetermined experimental endpoint in accordance with animal ethics requirements (defined as 365 days following implant).

### 3.3 TK-EPN862 histologically recapitulates the matched patient tumor

Histological assessment of tumor tissue from the TK-EPN862 PDOX (**Figure 5B**) demonstrated that the tumors growing in mice recapitulated many features of the patient tumor from which they were derived. Similar to the matched patient surgical sample (**Figure 4C**), TK-EPN862 tumors were highly cellular with evidence of perivascular pseudorosettes. Immunostaining of TK-EPN862 tumor cells was consistent with the features of EPN, including negative staining for OLIG2 and synaptophysin, positive staining for GFAP, and intracytoplasmic dot-like positivity for EMA. TK-EPN862 tumor cells were also negative for p53 and had a proliferative index of approximately 20%. Consistent with the features of PFA EPN, tumor cells from TK-EPN862 were negative for H3K27me3 and expressed high levels of EZHIP (**Figure 5B**). These histopathological features were maintained across *in vivo* passages of TK-EPN862.

### 3.4 TK-EPN862 molecularly classifies as PFA EPN

Methylation profiling of the first surgical sample from patient 801806 indicated it classified clearly as a PFA EPN (calibrated score >0.99 using the Molecular Neuropathology 2.0 classifier versions 11b4 and 12.5) (**Supplementary Table 1**). The metastatic surgical samples examined (two spinal lesions and one distal cranial lesion) also robustly classified as PFA EPN (calibrated score > 0.99 using the Molecular Neuropathology 2.0 classifier versions 11b4 and 12.5), irrespective of where the tumor recurred, consistent with the findings of others (4, 29). Additionally, the TK-EPN862 PDOX classified as PFA EPN (calibrated score > 0.98 using the Molecular Neuropathology 2.0 classifier versions 11b4 and 12.5), demonstrating faithful recapitulation of the original patient tumor in the mouse (**Supplementary Table 1**).

PFA EPN can be further divided molecularly into nine subtypes (PFA-1a-f and PFA-2a-c), each with distinct survival outcomes (10). Additional analysis of this patient’s disease using the most recent version of the Molecular Neuropathology 2.0 classifier (v12.5), which includes these subclasses, further classified all surgical and PDOX samples as PFA-2 EPN (calibrated score > 0.99). While the primary (first) surgical sample and the third surgical sample (spinal) were unable to be further subclassified, possibly due to normal tissue contamination, the second surgical sample and the PDOX robustly classified more specifically as PFA-2b (calibrated score >0.9). The fourth surgical sample from which the PDOX was derived also best classified as PFA-2b (calibrated score = 0.89) (**Supplementary Table 1**). Of note, PFA-2 tumors are associated with a higher rate of distant relapse compared to PFA-1 tumors (10), consistent with the features of this case.

### 3.5 Chromosomal abnormalities in the patient tumors increased with disease progression

**Figure 6:**
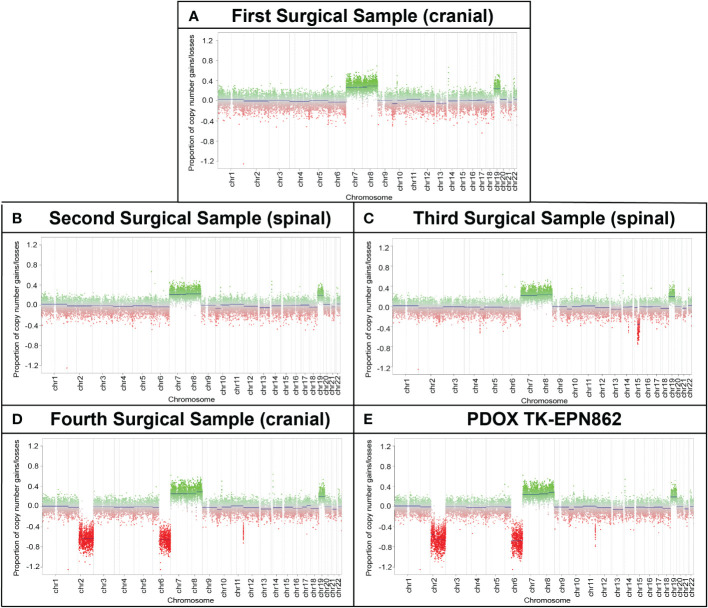
Longitudinal copy number analysis indicates the acquisition of additional genomic changes with disease progression. Copy number estimates (generated by MolecularNeuropathology.org using DNA methylation array data) for chromosomes 1 to 22 showing gains/amplifications (*green*) or losses (*red*) for **(A)** the primary cranial tumor (first surgical sample) and **(B, C)** two spinal metastases (second and third surgical samples). **(D)** The cranial metastasis (fourth surgical sample) and **(E)** TK-EPN862 PDOX (derived from the fourth surgical sample), exhibited the same chromosomal gains as samples 1-3, with additional losses of chromosomes 2q and 6q observed.

Copy number profiling using methylation data revealed gains of chromosomes 7, 8 and 19 across all surgical samples and in the TK-EPN862 PDOX tumor, supporting the notion that the secondary and subsequent tumors were metastases of the primary tumor, rather than *de novo* occurrences. Whole chromosome gains, including chromosomes 8 and 19 as observed in this case, are more common in PFA-2 EPN compared to PFA-1 EPN (10), and are consistent with the molecular classifications of the patient and PDOX tumors (**Supplementary Table 1**). In the fourth surgical sample and the matched PDOX, an additional loss of chromosomes 2q and 6q were observed, suggesting progressive genomic instability of the tumor (**Figure 6**). Gain of 1q, which is associated with more aggressive disease and poorer outcome in PFA EPN (4, 30), was not observed, consistent with the low frequency of this alteration in PFA-2b EPN (10).

**Figure 7:**
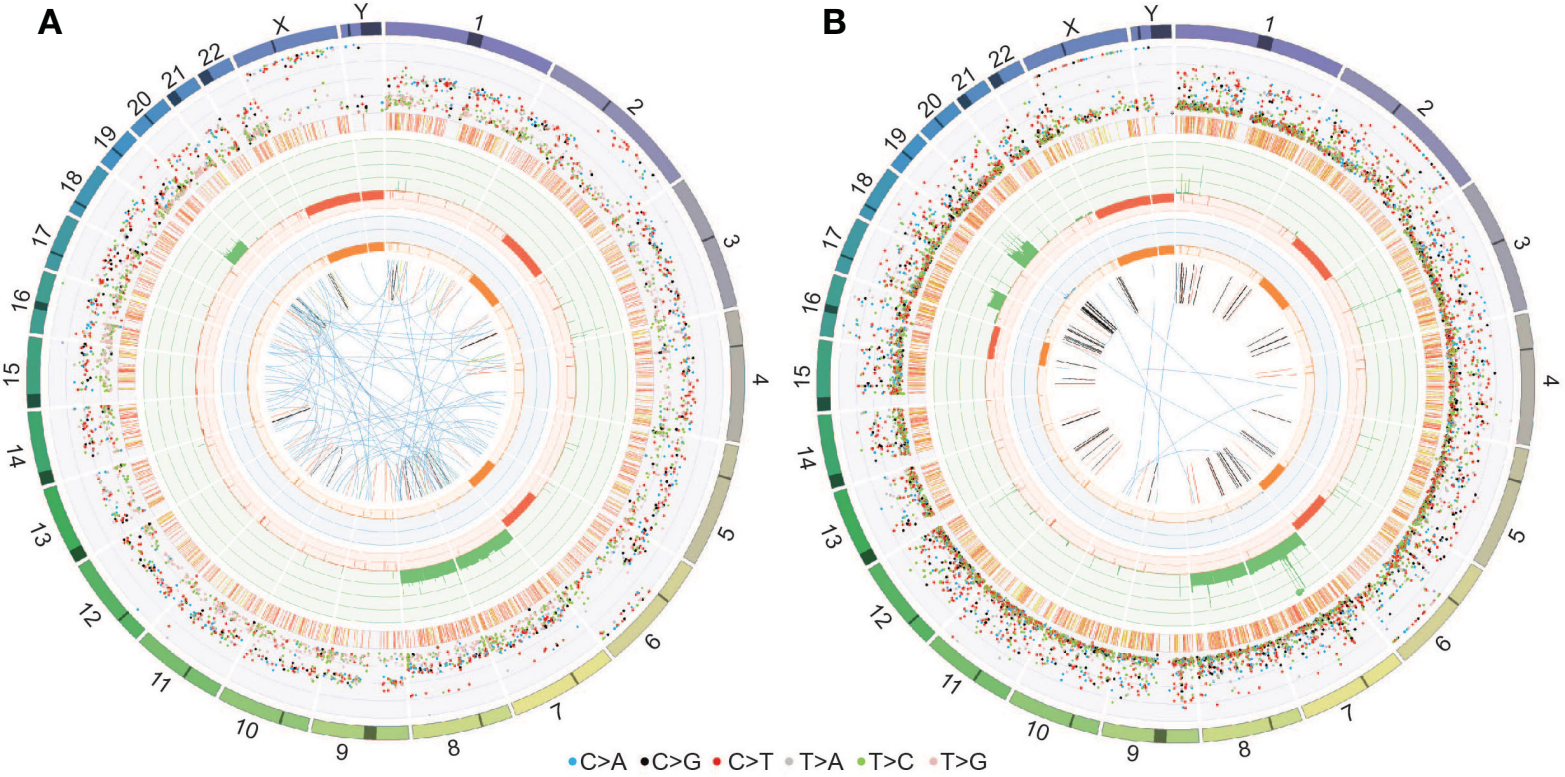
CIRCOS plots for **(A)** the fourth surgical sample and **(B)** the PDOX TK-EPN862 tumor confirm the chromosomal gains and losses observed in the copy number plots. Key to the CIRCOS plots: Outermost circle indicates the chromosomes, where darker shading represents large gaps in the human reference genome (e.g., centromeres). Second circle (*grey shading*) shows the somatic variants. These are divided into an outer ring of single nucleotide variants where each dot represents a single variant colored as shown with allele frequencies (corrected for tumor purity and scaled from 0-100%) and an inner ring of short insertions and deletions (*yellow* and *red*, respectively). Third circle (*red* and *green shading*) shows all observed tumor purity-adjusted copy number changes (losses and gains indicated in *red* or *green*, respectively; scale ranges from 0 (complete loss) to 6 (high level gains)). Fourth circle (*orange* and *blue shading*) represents the observed ‘minor allele copy numbers’ across the chromosome, ranging from 0 to 3. The expected normal minor allele copy number is 1. Values below 1 are shown as a loss (*orange*) and represents a loss of heterozygosity event, whilst values above 1 (*blue*) indicate amplification events of both alleles at the indicated locations. Innermost circle displays the observed structural variants within or between the chromosomes. Translocations are indicated in blue, deletions in red, insertions in yellow, tandem duplications in green and inversions in black.

WGS of the fourth surgical sample and matched PDOX confirmed the chromosomal abnormalities observed in the copy number plots (gain of chromosomes 7, 8 and 19 and loss of chromosomes 2q and 6q depicted in the third circle of the CIRCOS plots in **Figure 7**). In addition, loss of chromosome 16 and gain of 17q were observed in the PDOX by WGS (**Figure 7B**), however, no cancer-relevant genes in these locations were found to be significantly over- or under-expressed compared to the matched surgical sample by RNAseq. No single nucleotide variants of clinical significance were identified in either sample, consistent with the low mutation rates observed in PFA ependymomas (11). In particular, the absence of histone H3 K27M mutations [which are observed solely in PFA-1 EPN and absent from PFA-2 EPN (10)] are consistent with the molecular classification of these tumors as PFA-2 EPN.

### 3.6 Gene expression patterns were maintained across primary and metastatic tumors

**Figure 8:**
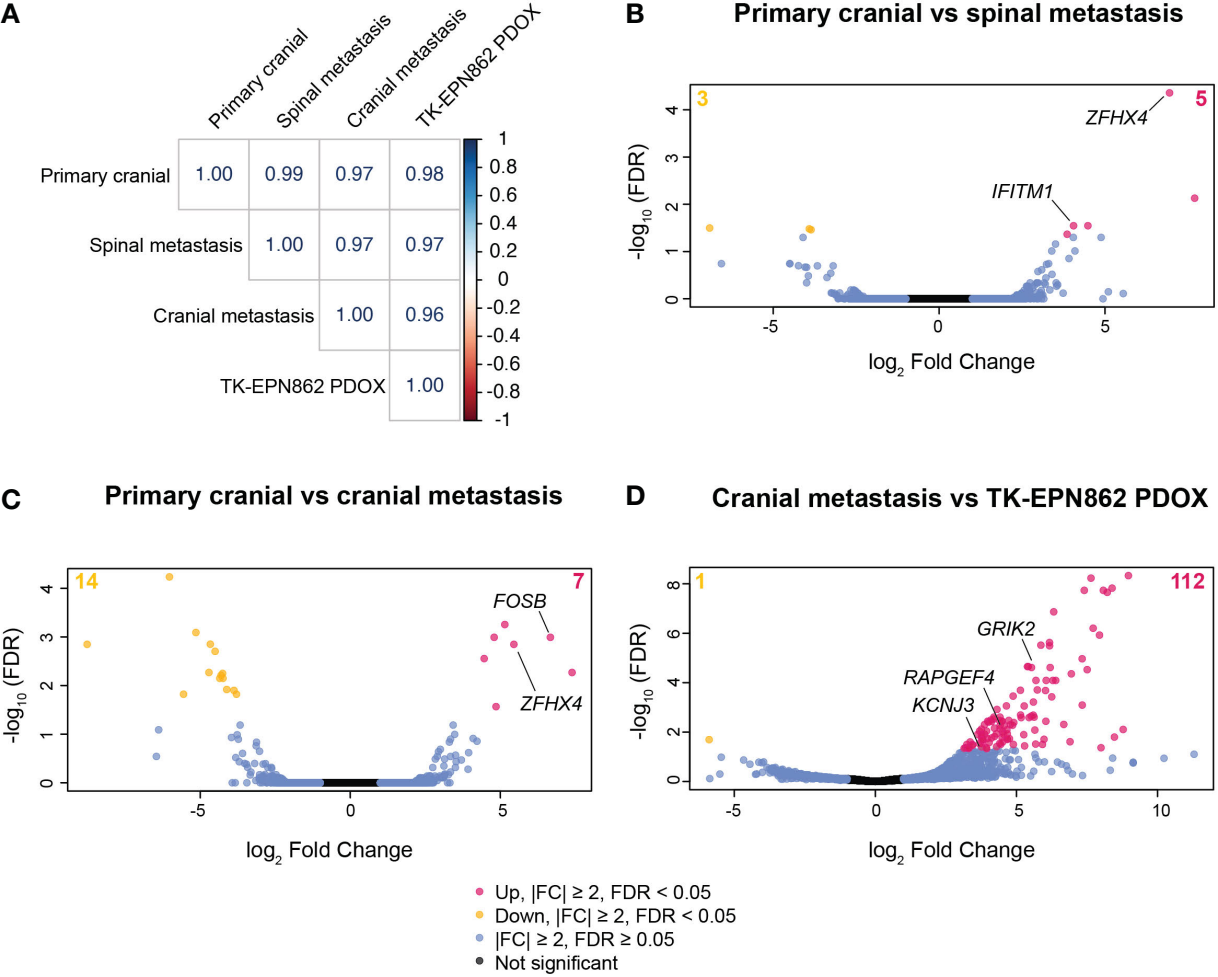
Gene expression patterns are highly conserved between the primary tumor, spinal metastasis, cranial recurrent lesion and the TK-EPN862 PDOX model. **(A)** Correlation matrix showing the correlation coefficient values for each sample comparison shown. **(B–D)** Volcano plots depicting differentially expressed genes between the samples stated on each plot. *Pink* dots and *yellow* dots represent genes that are statistically significantly over- or under-expressed, respectively, between the samples described (|FC| ≥ 2 and FDR < 0.05), *blue* dots represent genes that have |FC| ≥ 2 and FDR ≥ 0.05, and *black* dots represent genes that are not significantly differentially expressed (|FC| ≤ 2 and FDR ≥ 0.05).

In an effort to investigate if there were gene expression differences in the metastatic samples compared to the primary tumor that may provide new knowledge about relapsed EPN, we performed RNAseq on the primary tumor (first surgical sample), one subsequent spinal metastasis (third surgical sample), and the cranial recurrence (fourth surgical sample). There was insufficient high-quality RNA available from the second surgical sample to perform RNAseq on this tumor. Gene expression analysis showed little variance between the primary tumor, spinal metastasis and cranial recurrence, with correlation coefficient values above 0.95 between all sample comparisons (**Figure 8A**), despite the marked chromosomal losses observed in the fourth surgical sample compared to earlier samples. These data suggest that this PFA EPN predominantly retained its pattern of gene expression across metastases in different compartments of the CNS.

Comparing the primary tumor with one of the spinal lesions, eight genes were differentially expressed (**Figure 8B** and **Supplementary Table 2**), with *IFITM1* and *ZFHX4* being notable due to their described roles in cancer metastasis (31, 32). Expression levels of *IFITM1* increased 16.6-fold in the spinal metastasis compared to the primary cranial lesion (**Supplementary Table 2**). *IFITM1* is associated with glioma cell proliferation, migration and invasion (31), and so may have played a role in the metastatic process in this lesion. Additionally, the expression of *ZFHX4* increased over 120-fold in the spinal metastasis (**Supplementary Table 2**). Higher expression of this gene may have contributed to the progression of this tumor as *ZFHX4* has been associated with poor survival and metastasis in ovarian cancer (32) and is reported to play a role in the maintenance of tumor-initiating cells in glioblastoma (33).

When comparing the primary tumor and the cranial recurrence, 21 genes were significantly differentially expressed (|FC|≥2, FDR<0.05; **Figure 8C** and **Supplementary Table 3**), with expression of the proto-oncogene *FOSB* increased over 100-fold in the cranial recurrence compared to the primary tumor. *FOSB* has been reported to be highly expressed in glioma tissue compared to normal brain and is associated with glioma cell proliferation, migration, and invasion (34). The high expression levels of *ZFHX4* observed in the spinal metastasis were also observed in the cranial recurrence (43.8-fold increase compared to the primary tumor), reinforcing the possibility this gene may have played a role in the metastatic progression of this disease (**Supplementary Table 3**).

We next aimed to compare the transcriptome of TK-EPN862 with the matched lesion from which it was derived (the fourth surgical sample). Transcriptome analysis revealed 113 differentially expressed genes between the PDOX and the cranial metastasis (**Figure 8D** and **Supplementary Table 4**). We then performed KEGG pathway analysis using this gene list in order to elucidate specific biological pathways that may be altered in the PDOX. This revealed that most of the significantly altered genes were associated with pathways expressed in normal brain tissue (**Supplementary Table 5**). Additionally, three genes (*GRIK2*, *KCNJ3* and *RAPGEF4*) located on chromosomes 2q or 6q were highly overexpressed in the PDOX model, which was unexpected given the loss of 2q and 6q demonstrated by copy number estimates in both samples. Taken together, this suggests that the differential gene expression patterns observed are most likely due to normal mouse brain contamination, rather than alterations arising in the tumor cells post-engraftment.

**Figure 9:**
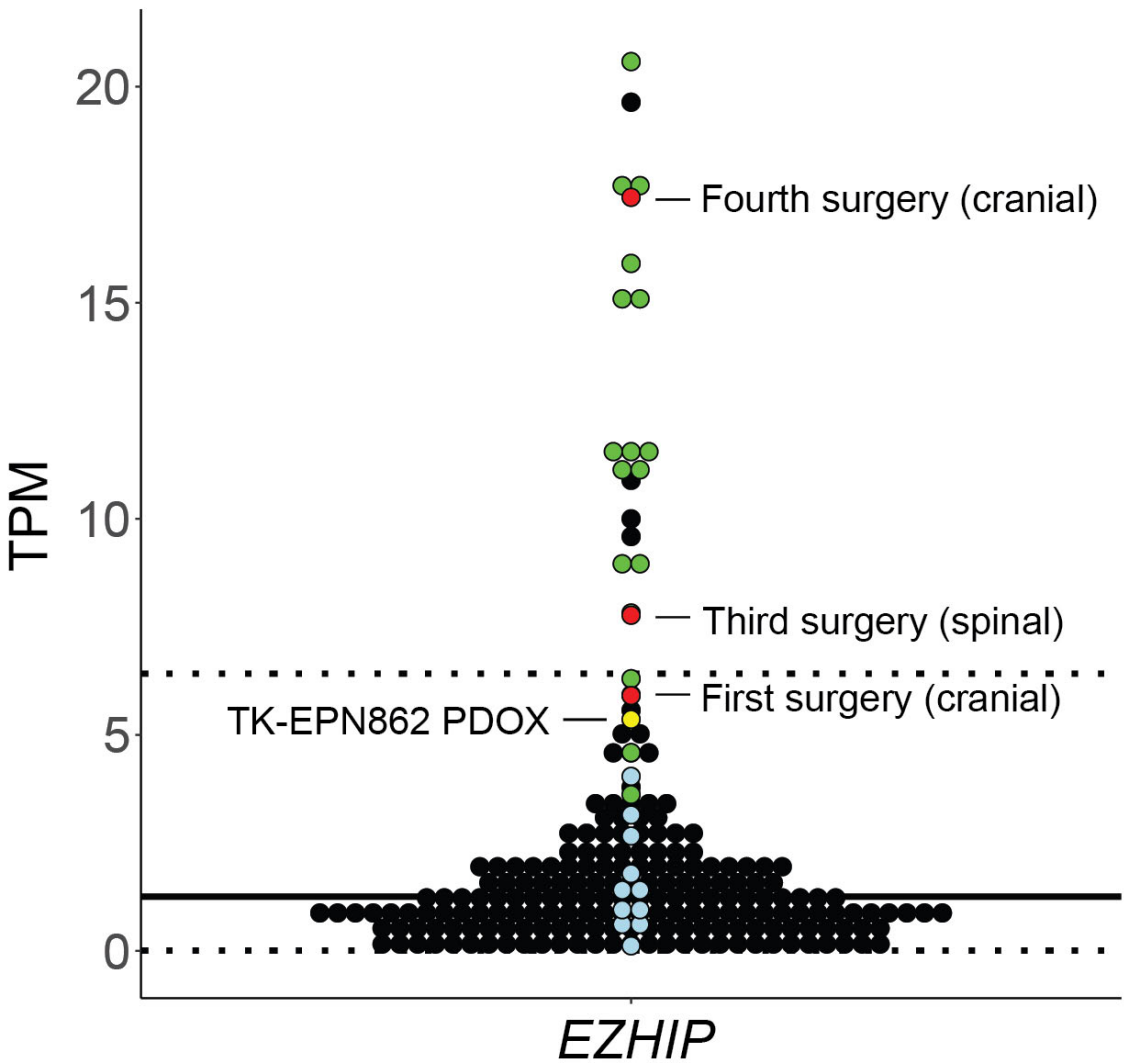
Gene expression levels (y axis: transcripts per million, TPM) for *EZHIP* in the first, third and fourth surgical tumor samples and matched PDOX TK-EPN862 tumor. The patient tumors (*red*) and TK-EPN862 (*yellow*) are compared to a reference cohort containing high-risk pediatric brain tumors. PFA EPN (*green dots*), other non-PFA EPNs (*blue dots*), and all other brain tumors (*black dots*) from the reference cohort are shown. Solid black line shows the mean TPM of the reference cohort, and dotted line shows the TPM values that are two standard deviations away from the mean.

Following this, we evaluated expression levels of *EZHIP* as a marker of PFA EPN (10), using the ZERO cohort of high-risk pediatric brain tumors as a reference dataset (25). As expected, PFA EPN within the reference cohort expressed high levels of *EZHIP* (**Figure 9**; green dots). High expression of *EZHIP* was observed in the first, third and fourth surgical samples, as well as in TK-EPN862 (**Figure 9**; red and yellow dots, respectively), which correlates with the high levels of EZHIP protein expression observed by IHC (**Figures 1**, **3**-**5**). The lower RNA expression level of the PDOX compared to the matched patient tumor (fourth surgical sample) is most likely due to the contaminating normal mouse brain tissue as discussed above.

### 3.7 1q gain may be an important predictor of PFA EPN PDOX establishment success

EPNs are challenging tumors to propagate in the mouse, with other laboratories publishing low success rates with this tumor type compared to other malignant CNS tumors (16, 18). Our combined data on attempts at establishing EPN PDOXs from the Telethon Kids Institute (Perth, Australia), Fred Hutchinson Cancer Centre (Seattle, USA) and Children’s Cancer Institute (Sydney, Australia) show that collectively only five out of 36 attempts (5/36; 13.8%) were successful beyond two passages *in vivo* (excluding pending attempts that have not yet had the opportunity to be propagated beyond two passages). Indeed, 25 of all attempts (25/39; 69.2%) failed to establish at all from the primary implant (**Supplementary Table 6**). Furthermore, of the five successful models, three of these have begun to demonstrate loss of tumorigenicity at later *in vivo* passages, further highlighting the challenges of creating EPN PDOX models.

As the development of these models requires significant time and resource input for a relatively low chance of engraftment success, we sought to identify any biomarkers that may be indicative of increased likelihood of PFA EPN PDOX generation success. Despite the aggressive nature of the tumor in the patient presented in this report, the tumor and the matched PDOX did not demonstrate 1q gain, which is associated with poorer outcomes and more aggressive disease in PFA EPN (4, 30). Of the published PFA EPN PDOX models with molecular data, 1q gain was reported in all but one of these models (16, 18, 35), raising the possibility that 1q gain may be associated with an increased likelihood of a PFA EPN PDOX successfully establishing. To investigate this, we performed DNA methylation array to determine 1q status on the tumors from all historic attempts to establish a PFA EPN PDOX model in the laboratories of Telethon Kids Institute (Perth, Australia) and Children’s Cancer Institute (Sydney, Australia). A lack of primary patient material precluded analysis on unpublished samples from the Fred Hutchinson Cancer Center cohort. Molecular classification as PFA EPN were confirmed for all tumors using the Molecular Neuropathology 2.0 classifier (v11b4 and v12.5). A successful PDOX model was defined as having been successfully propagated beyond two passages *in vivo*. Including data from published PDOX models, we found that 10/11 successful PFA EPN PDOX models had 1q gain, compared to only 1/7 attempted PFA EPN PDOX models that failed to establish (**Table 1** and **Supplementary Figure 2**), and that this difference was statistically significant (p = 0.0025). Although the sample size is small owing to the rarity of this specific subtype, these data suggest that 1q gain may be an important predictor of PFA EPN PDOX establishment success.

**Table 1:** 1q gain is associated with an increased likelihood of establishment success of PFA EPN PDOX models.

Institute	Sample ID	Primary implant established in mouse	Status*	1q status	Other genetic alterations reported	Publication
Fred Hutchinson Cancer Center	EPD-210FH	Y	Successful	1q gain	Chr 6q loss. Chr 10q loss. Chr 11q loss. Chr 12p loss. Chr 17 gain. Chr 22q loss.	(16, 36)
Fred Hutchinson Cancer Center	EPD-710FH	Y	Successful	1q gain	Chr 10 loss.	(16, 36)
Children’s Hospital Colorado/University of Colorado	MAF-811_XC	Y	Successful	1q gain	High expression of *EZHIP*. Chr 6 loss. Chr 22 loss.	(35)
Children’s Hospital Colorado/University of Colorado	MAF-928_XC	Y	Successful	1q gain	High expression of *EZHIP*. Chr 6 loss.	(35)
St Jude Children’s Research Hospital	SJEPPF-15-8710	Y	Successful	1q gain	High expression of *EZHIP*. Low H3K27me3 methylation. Chr 6q loss. Chr 10q loss.	(18)
St Jude Children’s Research Hospital	SJEPPF-16-02472	Y	Successful	1q gain	High expression of *EZHIP*. Low H3K27me3 methylation. Chr 9 gain.	(18)
St Jude Children’s Research Hospital	SJEPPF-16-08404	Y	Successful	Balanced	High expression of *EZHIP*. Low H3K27me3 methylation. *APOB* mutation. *CDKN1B* and *CDKN2C* mutations. Chr 6q loss. Chr 16q loss.	(18)
St Jude Children’s Research Hospital	SJEPPF-16-09238	Y	Successful	1q gain	High expression of *EZHIP*. Low H3K27me3 methylation. *RAG1* mutation. Chr 16q loss. Chr 22q loss.	(18)
Baylor College of Medicine	0614EPN	Y	Successful	1q gain		(29)
Baylor College of Medicine	2002EPN	Y	Successful	1q gain		(29)
Baylor College of Medicine	4423EPN	Y	Successful	1q gain		(29)
Telethon Kids Institute	801806/TK-EPN862	Y	Failed	Balanced	High expression of *EZHIP*. Low H3K27me3 methylation. Chr 2q loss. Chr 6q loss.	Model described in this report
Telethon Kids Institute	857224	N	Failed	Balanced	No whole arm chromosomal alterations found.	Unpublished
Telethon Kids Institute	861048	N	Failed	1q gain	Chr 16q loss.	Unpublished
Telethon Kids Institute	861756	N	Failed	Balanced	No whole arm chromosomal alterations found.	Unpublished
Telethon Kids Institute	903149	N	Failed	Balanced	No whole arm chromosomal alterations found.	Unpublished
Telethon Kids Institute	906462	N	Failed	Balanced	No whole arm chromosomal alterations found.	Unpublished
Children’s Cancer Institute	P001001	N	Failed	Balanced	High expression of *EZHIP*.	Unpublished
Children’s Cancer Institute	P002103	Y	Pending	1q gain	High expression of *EZHIP* and *SMYD3*. Low expression of *CDKN1A:SH2B3.*	Unpublished
Children’s Cancer Institute	P012301	Y	Pending	1q gain	High expression of *EZHIP*, *HSP90AA1*, *ABL2*, and *VEGFA.*	Unpublished

1q status of all attempts to establish PFA EPN PDOX models from Telethon Kids Institute and Children’s Cancer Institute as well as published data are shown. Other reported genetic alterations including chromosome (Chr) loss or gain are described. *Status categories are as follows: Successful - sustained propagation of PDOX beyond two passages *in vivo*; Failed - PDOX did not propagate from the primary implant or failed to propagate beyond two passages *in vivo*; Pending - PDOX still being established and has not yet been propagated past two passages *in vivo*.

## 4 Discussion

PFA EPN is one of the deadliest brain cancers in children. Here, we describe the case of a patient that presented with a cranial PFA EPN that later metastasized multiple times to the spine. The cancer then recurred at a distal site in the brain before the patient succumbed from widely disseminated metastatic disease through the CNS. Transcriptome analyses demonstrated significant similarity between the primary tumor and the spinal and cranial metastases, suggesting these recurrent lesions had not genetically diverged from the primary lesion. The most notable genes that were significantly overexpressed in the metastases compared to the primary tumor (*IFITM1*, *ZFHX4* and *FOSB*) are associated with glioma proliferation, migration and invasion (31, 34), and maintenance of glioblastoma tumor initiating cells (33), suggesting they may have contributed to the progression and metastasis of this disease. This is in contrast with a recent study, where expression of a different subset of genes including *NOTCH*, *EPHA2* and *SUFU* were reported to be significantly altered in metastases of pediatric PFA EPN compared to the primary tumor (29). Longitudinal primary and relapse samples from pediatric PFA EPN patients are very rare, with Zhao and colleagues (29) reporting on just five patients with matched primary and metastatic tumors over a 13-year period. Consequently, it is possible that the differences in genes reported may be due to the small sample size examined in each study, highlighting the need for further research in a larger number of longitudinal patient samples.

From the cranial recurrence, we generated and characterized a PFA EPN PDOX model, TK-EPN862, that faithfully recapitulated the matched patient tumor from which it was derived. Despite the aggressive nature of the tumor in the patient, the PDOX was unable to be maintained past two passages in mouse brain before losing tumorigenicity. In all but one PFA EPN PDOX models published with molecular data, high expression levels of *EZHIP* and 1q gain were reported (16, 18, 35). The one model that did not have 1q gain harbored additional alterations including mutations in *APOB*, *CDKN1B* and *CDKN2C*, potentially driving tumorigenicity (18). *EZHIP* overexpression at both the RNA and protein level is characteristic of PFA EPNs, with the exception of the PFA-1f subtype (10). Overexpression of *EZHIP* inhibits polycomb repressive complex 2 function, resulting in the global reduction of H3K27me3 in PFA EPN (37), and is mutually exclusive with histone H3 K27M mutation (38). In TK-EPN862 and the matched patient tumor, we observed high expression of *EZHIP* RNA and protein, and the associated low levels of H3K27me3 detected *via* IHC, with a lack of histone gene mutations, consistent with a diagnosis of PFA EPN. However, whilst high levels of *EZHIP* expression were observed in TK-EPN862, there was no evidence of the 1q gain consistently reported in successful PDOX models of PFA EPN. Indeed, in combination with published data, retrospective analysis of our attempts to establish PFA EPN PDOX models demonstrated that PFA EPN tumors that harbor 1q gain are more likely to lead to successful PDOX establishment than tumors that do not (**Table 1**). In support of this theory, Zhao and colleagues (29) recently demonstrated that 1q gain in primary PFA EPNs is consistently maintained upon orthotopic xenograft, supporting a role of 1q gain in the tumorigenicity of this disease. As 1q gain is associated with poorer outcomes and more aggressive disease in PFA EPN (4, 30), it is possible that the lack of this alteration (in the absence of other oncogenic alterations) in TK-EPN862 contributed to its inability to be serially transplanted *in vivo* beyond two passages.

Although chromosome 1q was unaltered, TK-EPN862 PDOX and its matched patient tumor harbored a number of large-scale copy number alterations including gains in chromosomes 7, 8 and 19, and loss of 2q and 6q. A recent study of 240 pediatric PFA EPN reported that while gain of either chromosome 7 (12/240), 8 (15/240) or 19 (12/240) were observed in 5-6% of PFA EPN tumors, few demonstrated concurrent gain of all three chromosomes (2/240), and loss of 2q was rarely observed (2/240) (4). A more recent analysis showed that whole chromosome gains, including gain of chromosomes 8 and 19 were more common in PFA-2 EPNs (as is the case described here) compared to PFA-1 EPNs (10). These findings suggest that these alterations may be recurrent in this specific subset of PFA EPN, although their significance in the development or progression of these tumors remains unclear. Whilst whole chromosome 7 gain has been associated with an increased risk of recurrence in pilocytic astrocytomas (39), this link in PFA EPN has not yet been demonstrated. By contrast, 6q loss was more frequently observed in PFA EPN (25/240) in the Pajtler et al. (4) analysis and has been associated with recurrence in PFA tumors and poor prognosis independent of 1q gain (10, 12, 40, 41).

No clinically significant mutations were present in TK-EPN862 or the matched patient tumor. Unlike some other brain tumor types, PFA EPNs are often genetically silent and lack hallmark gene amplification or specific recurrent mutational events (11, 42). Instead, PFA EPNs tend to demonstrate global changes in the epigenome, with widespread loss of histone H3 K27 tri-methylation being the major tumor driver (9, 10). Efforts to mimic such events in the laboratory to genetically engineer mouse models of PFA EPN is challenging. This is in contrast to the development of mouse models for supratentorial EPN, where expression of the *ZFTA-RELA* fusion is strongly tumorigenic (42–44). Overexpression of *EZHIP* in mouse hindbrain progenitor cells has been shown to generate tumors that resemble EPN in the mouse (45); however, this required additional genetic alterations not common in PFA EPN.

Given these challenges, PDOX models would be incredibly valuable for PFA EPN translational research; however, as our study highlights, the success rate of establishing such models in the laboratory is low. Others have also noted lower success rates for this tumor subtype compared to all other CNS malignancies attempted, including medulloblastoma, primitive neuroectodermal tumors, atypical teratoid/rhabdoid tumors, and high-grade gliomas (16, 18), demonstrating how difficult these models are to generate. Even in laboratories that have had success generating EPN PDOX models (16), the gradual loss of tumorigenicity with subsequent *in vivo* passages is not uncommon, highlighting the challenges of generating PDOX models of this particular brain tumor type.

One possible reason for the lack of PDOX success for PFA EPN is the potential role of the tumor microenvironment, which is becoming increasingly important in our understanding of these cancers. Preliminary data suggest that PFA EPN cell proliferation and tumor progression may be driven by a cycle of continual and unresolved “wound repair”, initiated by hypoxia or myeloid cell interactions that trigger epithelial-mesenchymal transition (46). Indeed, Michealraj et al. (47) demonstrate that primary cultures of PFA EPN grow best in hypoxic conditions (1% oxygen), where they have a higher establishment rate, proliferate more, and have reduced markers of cellular senescence and apoptosis. Hypoxia also plays a critical role in the characteristic hypomethylation of lysine 27 on histone H3 in PFA EPN (47). This group went on to report that hypoxia gene expression signatures are at their peak in the murine fetal hindbrain microenvironment at the same point in development when the cells of origin for PFA EPN arise, specifically embryonic days (E) 10 and 16 in the mouse (47–49). Additionally, the metabolic phenotype of mouse hindbrain at E16 closely resembles that observed in PFA EPN (47).

In this study, we exclusively used adult immune-deficient mice to propagate PDOXs. Based on the findings of Michealraj et al. (47), we hypothesize that implantation of patient-derived PFA EPN cells into embryonic mouse brains at approximately E16 may improve PDOX success, as this coincides with conditions in which the microenvironment is most supportive of PFA EPN growth. Whilst the use of immune-compromised strains is common for PDOX modelling, there have been reports of successful intracranial implantation of patient-derived glioblastoma cells into immune-competent E12.5 mice (50). Although the number of tumor-bearing brains progressively decreased after birth, tumors persisted in some mice at P28, highlighting the exciting potential of this technique. If established, an embryonic PDOX model in an immune-competent mouse such as that described in Hoffmann et al. (50) would also allow investigation of immune cell interactions in the development and treatment of PFA EPN.

In conclusion, PFA EPN is the most common and the deadliest subclass of EPN in children, with high rates of recurrence. There is a pressing need for more effective treatments for these patients. PDOX models facilitate a better understanding of the biology of the disease and allow for preclinical testing of novel therapies, with the hope of translation to the clinic and improved outcomes for patients. The development of PDOX models of PFA EPN is urgently needed and very challenging. We have extensively characterized a PDOX model of PFA EPN that persisted *in vivo* for two passages before losing tumorigenicity. Comparison with successful models developed across six independent laboratories suggests that 1q gain, predictive of tumor aggression and poor outcome clinically, may be an indicator of likely PDOX generation success. Additionally, we postulate that implantation of patient-derived tumor tissue into the brains of embryonic mice may increase the chances of success, as the microenvironment is most supportive of PFA EPN tumor growth at this stage of development.

## Data availability statement

The datasets presented in this study can be found in online repositories. The names of the repositories and accession numbers can be found below: EGA archive, WGS: EGAS00001006843, RNAseq: EGAS00001006844.

## Ethics statement

The studies involving human participants were reviewed and approved by the Ethics Committee of the Child and Adolescent Health Service, Western Australia (HREC: 1769/EP (PRN 0000002372) A Perth Children’s Hospital Oncology Protocol for Collecting and Banking Paediatric Research Specimens. Written informed consent was obtained from the minor’s legal guardian for the publication of any potentially identifiable images or data included in this article. The animal study was reviewed and approved by the Animal Ethics Committee of the Telethon Kids Institute and performed in accordance with Australia’s Code for the Care and Use of Animals for Scientific Purposes.

## Author contributions

Conceptualization, JW, NG, RE. Methodology, JW, HH, CM, MW, MC, CW, MT, EG, RE. Software, MC, CM, MW. Formal analysis, JW, MH, CM, MW, PB, PA, LC, RE. Investigation and validation, JW, HD, HH, CM, MW, PB, PA, LC, CW, MB, JB, KR, MT, EG, JD, SL. Resources, RE, NG, PE, MC, SL, JD, MT, EG, DZ. Data curation, JW, HD, CM, MW, PB, PA, LC, MC, PE, RE. Writing—original draft preparation, JW, HD, MH, RE. Writing—review and editing, all authors. Visualization, JW, HD, MH, RE, CM, MW, PB, PA, LC. Supervision, NG, RE, MC, PE. Project administration, RE, JW. Funding acquisition, NG, RE, MC, PE, DZ. All authors contributed to the article and approved the submitted version.
