# Supplementary material for: In vivo loss of tumorigenicity in a patient-derived orthotopic xenograft mouse model of ependymoma
Source: Front Oncol. 2023 Mar 3;13:1123492. doi: 10.3389/fonc.2023.1123492 (PMC10020925; doi:10.3389/fonc.2023.1123492)
Supplement: Supplementary file 1 [file DataSheet_1.pdf]

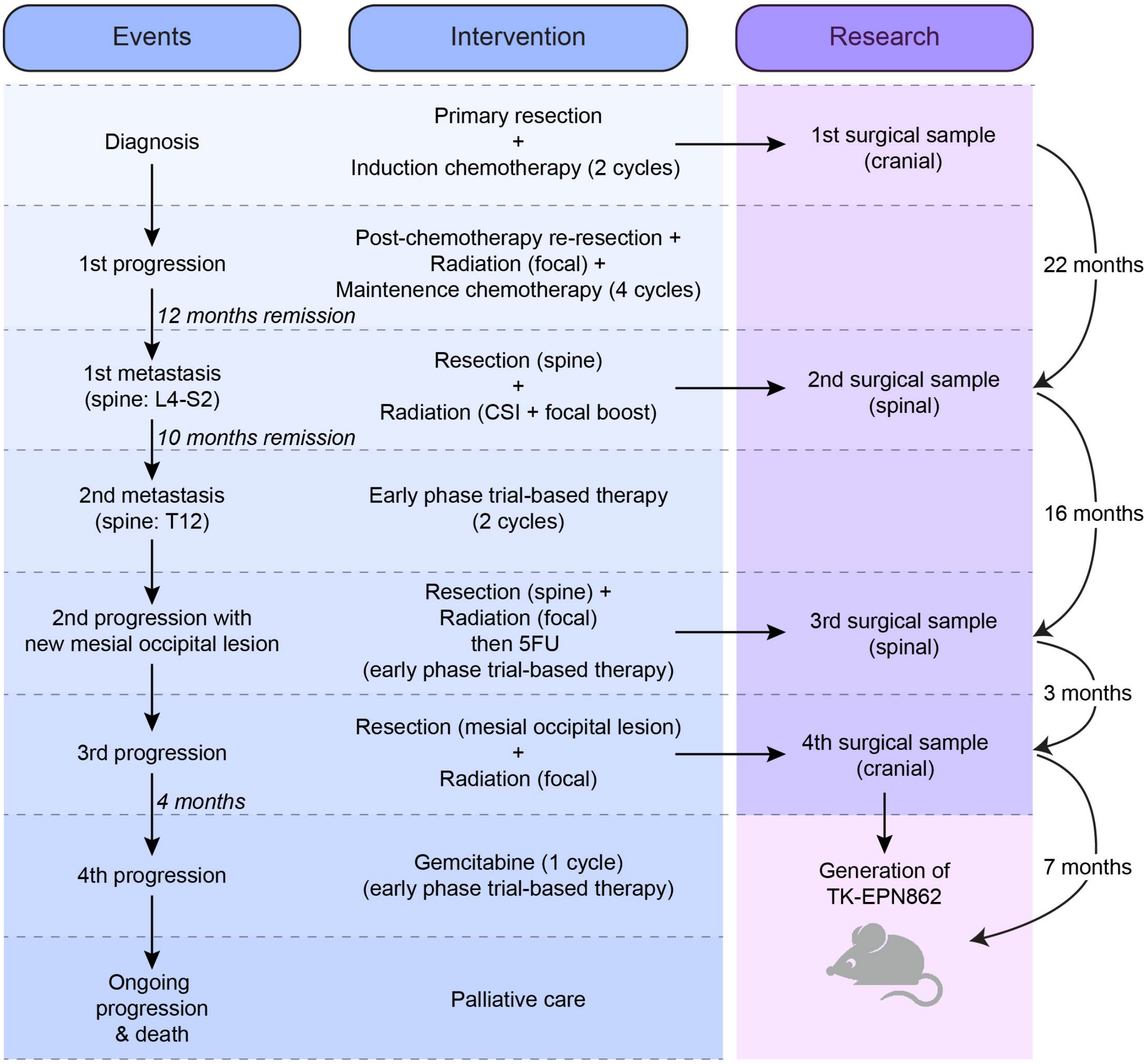

Supplementary Figure 1: Timeline depicting clinical events, treatment interventions and surgical samples collected for histological and genetic analysis. Abbreviations: CSI, craniospinal irradiation; 5FU, fluorouracil
