# Supplementary material for: In vivo loss of tumorigenicity in a patient-derived orthotopic xenograft mouse model of ependymoma
Source: Front Oncol. 2023 Mar 3;13:1123492. doi: 10.3389/fonc.2023.1123492 (PMC10020925; doi:10.3389/fonc.2023.1123492)
Supplement: Supplementary file 2 [file DataSheet_2.pdf]

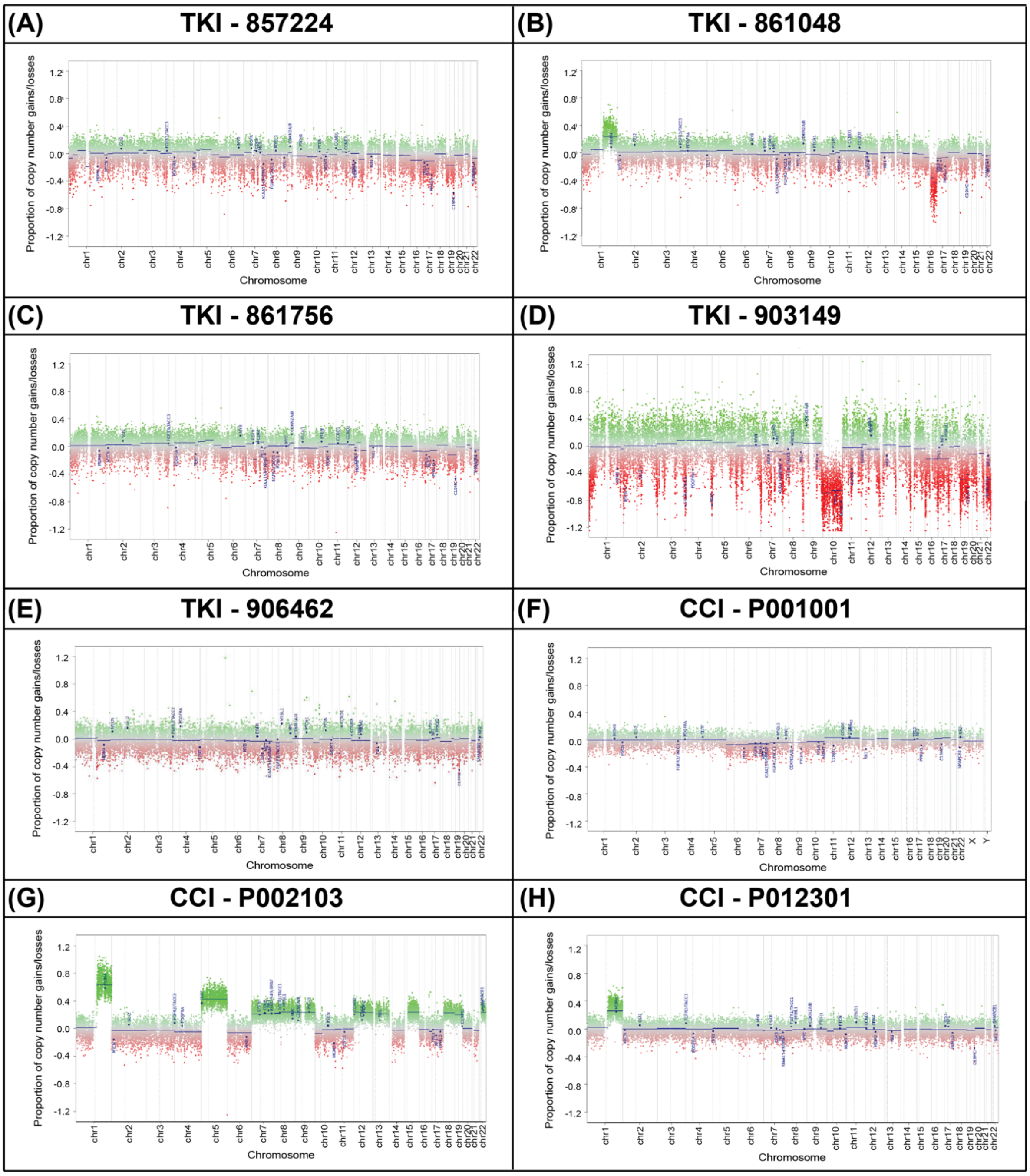

Supplementary Figure 2: Copy number plots generated from DNA methylation analysis of PFA EPN primary tumors from which PDOX establishment was attempted. Brain tumor relevant genes (n=29) are automatically annotated. **(A-F)** represent tumors that failed to establish as PDOX models. These tumors do not have 1q gain. **(G-H)** represent tumors that have generated tumors in mice but have not yet been passaged more than two times *in vivo* (pending). These tumors have 1q gain. ID numbers for each tumor are shown. TKI = PDOX attempted at Telethon Kids Institute (Perth, Australia). CCI = PDOX attempted at Children's Cancer Institute (Sydney, Australia).
